# Effect of three natural antioxidants on the structure and physicochemical properties of sweet potato starch noodles

**DOI:** 10.3389/fnut.2022.1020281

**Published:** 2022-10-28

**Authors:** Weiyun Guo, Ling Fan, Yonghui Wang, Guanghui Li, Xueli Gao, Zhenhao Chen, Jihong Huang

**Affiliations:** ^1^Food and Pharmacy College, Xuchang University, Xuchang, China; ^2^College of Agriculture, Henan University, Zhengzhou, China

**Keywords:** sweet potato starch noodles, curcumin, tea polypheno, lycopene, antioxidation capacities, structure and physicochemical properties

## Abstract

The study aimed to investigate the effect of three kinds of natural antioxidants (NAs), such as curcumin, tea polyphenols (TP), and lycopene, on sweet potato starch's structure and physicochemical properties of starch noodles. We found that the broken rates, iodine blue values, hardness, and chewiness of natural antioxidant starch noodles (NASN) were increased with the addition of the NAs. Additionally, the elasticity decreased with the addition of curcumin and lycopene, but it increased with the addition of TP. The cross-section structure of NASN obtained by scanning electron microscope (SEM) showed more holes appeared when adding NAs, and the additional amount had a pronounced effect on the microstructure of starch noodles (SN) regardless of the kind of NA added. The X-ray diffraction detection showed that some crystal forms were significantly damaged, and the addition of NAs affected the crystallization process of starch and produced a small proportion of new crystals in the NASNs. The protective effects of SN on NAs and their antioxidant capacities under dry and room temperature storage (DRTS) and wet and frozen storage (WFS) conditions were optimal as compared to those of flour noodles (FN). The results showed that adding NAs could improve the sensory quality and antioxidant function of starch noodles. In turn, the dense structure of starch noodles can also have a significant protective effect on antioxidants and their antioxidant activities, which is especially obvious under WFS conditions.

## Introduction

The sweet potato output in China ranks first in the world, accounting for more than 85% of the world's total output. The glycemic index (GI) of sweet potato is <55, which is a typical low GI healthy food raw material (1, 2). In China, 28% of sweet potato products are processed into starch noodles (SN), a food popular in many Asian countries, including China, South Korea, Vietnam, and Indonesia (3, 4). SN, which is made of tuber starch (such as cassava or sweet potato), does not contain gluten and is very suitable for people with celiac disease or gluten intolerance (2). However, from the perspective of nutrition or functionalization, SN lacks protein, vitamins, minerals, polyphenols, cellulose, and other functional components, and their comprehensive nutritional value does not perform high. Therefore, it is necessary to improve the quality of SN to make their nutritional value more comprehensive.

The improvement of the quality of SN mainly focuses on food quality, nutrition improvement, and functionalization. In some studies, by adding concentrated whey protein, banana starch, and NUTRIOSE^®^ (resistant starch) to sweet potato starch noodles (SPSN), the protein retention rate and resistant starch content in the products could be effectively improved, which could also reduce the *in vitro* starch digestibility (2, 5). Additionally, using antioxidants in foods is necessary to regulate health through diet. Oxidative stress is caused by an imbalance in the production of reactive oxygen species and the biological ability to detoxify the reactive intermediates or repair the resulting damage (6). Importantly, antioxidants are substances that remove, delay or prevent oxidative damage, and they can control the amount of oxygen free radicals to neutralize oxidative damage and thus protect the body (7). Curcumin, for example, can reduce the effects of chronic inflammation on human liver cells and consistently relieve arsenic-induced elevation of serum alanine aminotransferase and aspartate aminotransferase activities, augment hepatic malonaldehyde, and reduce blood and hepatic glutathione levels (8). Additionally, Lan et al. studied clinical wound treatment and found that tea polyphenols showed sustained release through *in vitro* digestion, which was conducive to the realization of immediate bacteriostatic effects in the initial stage of wound healing and long-term antioxidant activity (9)_._ Furthermore, lycopene can protect human somatic cells from free radical damage, enhancing the body's ability to fight disease, delay aging, and resist cancer (10). Therefore, polyphenols have antibacterial, anti-cancer, anti-diabetes, immune regulation, anti-atherosclerosis, kidney protection, and other beneficial effects (11).

Considering these findings, adding substances with antioxidant function to starch products could effectively improve their stability, antioxidant properties, and functional quality. For example, adding quercetin and Tert-butyl hydroquinone (TBHQ) to cassava starch/gelatin composite film increased the film's water solubility and water vapor permeability and effectively delayed the oxidation of lard through the film (12). In some studies, finger millet added to rice noodles can not only effectively guarantee the quality of the product, such as crushing rate, cooking loss rate, protein content, and dietary fiber, but also significantly improve the antioxidant activity of the product and its 1,1-diphenyl-2-trinitrophenylhydrazine (DPPH) scavenging ability by 169.6%. Additionally, adding matcha powder to rice noodles effectively reduced the digestibility of starch, increased the content of resistant starch, reduced the GI value, and improved the product's antioxidant and flavor. Moreover, the addition of matcha powder interferes with the recombination of starch chains, resulting in the formation of low-ordered structures, which would decrease the glycemic index of the noodle (13). Likewise, adding cedar leaf powder to rice noodles effectively increased the total phenol content and improved the scavenging ability of the DPPH-free radical (14). Similarly, adding Green Mussel to gluten-free pasta significantly increased the product's ionic strength and gelation level, as well as the free radical scavenging and reducing capacity (15). Notably, these investigations had a high reference value for the quality improvement of SN. Furthermore, there have been many attempts to improve the quality of SN. For example, some researchers added pumpkin powder to SNs, which had no adverse effects on their aroma and taste, but effectively improved the antioxidant activity of flat potato noodles (16). When mulberry leaf powder was added to SN, it was found that it significantly increased the mineral content and total phenol content in SNs, and the antioxidant, anti-diabetic, anti-hypertensive, and anti-diabetic activities of starch vermicelli were improved considerably (17). Therefore, it is speculated that adding NAs to SN may not only ensure the quality but also improve the nutritional and antioxidant properties of NASN.

This study mainly prepared natural antioxidants starch noodles (NASNs) and investigated the effects of natural antioxidants, such as curcumin, TP, and lycopene on the broken rate, iodine blue value, sensory quality, texture characteristics, and structure of SPSN. In addition, the protective effects of NAs and oxygen-free radical scavenging function were studied under two storage conditions: dry and room temperature storage (DRTS) and wet and freezing storage (WFS). Furthermore, the interaction between NAs and SPSNs was discussed to lay a foundation for developing starch noodle products with certain physiological functions.

## Materials and methods

### Reagents and materials

Sweet potato starch (SPS) (Grade II) with an amylose proportion of 27.5% was purchased from the Shandong Shengqi Biological (Jining, China). High-gluten wheat flour was purchased from One Plus One Natural Flour (Zhengzhou, China). Food-grade curcumin and TP with a purity of 95 and 98%, respectively, were purchased from the Youbaojia Food Co., Ltd (Shangqiu, China), and the food-grade lycopene with a purity of 96% was purchased from the Shengjiade Biotechnology Co., Ltd (Qufu, China). Folin reagent with a concentration of 1 mol/L was purchased from Bomei Biotechnology (Hefei, China). Anhydrous sodium carbonate was purchased from the Kaitong Chemical Reagent (Tianjin, China). N-hexane with a purity of 99.5% and pure analytical-grade anhydrous ethanol was purchased from the Xianshuigu Industrial Park (Tianjin, China). Toluene with a purity of 99.5% and acetic acid with a concentration of 1 mol/L were purchased from the Tianjin Kermel Chemical Reagent (Tianjin, China). DPPH, with a purity of 98%, was purchased from Shanghai Yuanye Biotechnology (Shanghai, China).

### Preparation of NASNs

Seventy-five grams of sweet potato starch were accurately weighed and placed in a 1,000-ml beaker. We added 0.15 g of TP or lycopene or 0.20 g of curcumin into the beaker, heated it to 90°C using an electric constant temperature water bath (Putian, China, Dk-8d), and stirred for 3 min to make the starch uniform. After gelatinization, we added 25.0 g of dry sweet potato starch into the gelatinized starch and stirred it evenly. We then mixed the dough at 40°C for 6 min on a smooth surface, put it in an extruder, and kept the extrusion process at a uniform speed to prevent indentation. We quickly cut the strips into the water above 90°C for cooking until they were transparent, then removed them and immediately put them into cold water for cooling. Finally, we hung them on the noodle rack for drying and packed them after 24 h to make dry NASNs. We also directly packaged them without any dry process and froze them to make frozen NASNs.

### Preparation of natural antioxidant flour noodles

To compare and verify the protective effects of sweet potato SNs on NAs and antioxidant activity under different storage conditions, natural antioxidant flour noodles were prepared as the control group. We accurately weighed 100 g of flour, added 0.15 g of TP or lycopene or 0.20 g of curcumin, and 40 ml of purified water, stirred, and kneaded it to form a dough with a smooth surface, and put it in the extruder for extrusion. The extruded noodles were hung on the noodle rack for drying and packaged after 24 h. Finally, wet noodle samples were packaged directly and frozen in the refrigerator's freezer.

### The effect of NAs addition amount on the quality of NASNs

To determine the optimal parameter level of the addition amount of NAs, the broken rate, paste soup rate, sensory evaluation, and texture characteristics of NASNs were determined. The addition amounts of NAs were selected as 0.05, 0.10, 0.15, 0.20, and 0.25 g and then made into SNs. Broken rates, iodine blue values, sensory scores, and TPA texture characteristics were selected as proxies to determine the optimal addition amounts of the NAs.

### Analysis of broken rate of NASNs

A total of 20 pieces of 10 cm long NASNs were made and soaked in cold water for 5 min until fully swollen. They were taken out and put into 1,000 ml of distilled boiling water for 30 min. The noodles were drained, and water was filtered out using absorbent paper. We then counted the total number of broken noodles and calculated the percentage of broken strips in the total number of noodles.

### Analysis of iodine blue value of NASNs

After boiling the NASNs for 30 min, the soup was cooled to about 20°C. Five milliliters of cooled soup was poured into a 10-ml volumetric flask, and 1 ml of acetic acid solution was added for a concentration of 1 mol/L and shaken. Then, we added 1 ml iodine reagent for a concentration of 0.1 mol/L, followed by adding deionized water to the scale. The sample was then shaken, followed by stranding for 5 min. The absorbance of the sample solution was determined by a spectrophotometer (Xinmao, China, UV-7504) at the wavelength of 620 nm with distilled water as blank.

### Analysis of sensory score of NASNs

Five trained sensory evaluators were selected to make a sensory evaluation on different noodles. To evaluate the pre-cooking characteristics of the product, texture (25%), including thickness, elasticity, adhesiveness, and color (25%), was analyzed before boiling. Furthermore, odor, taste (25%), and impurities (25%) were analyzed after boiling.

### Textural profile analysis

Fifteen NASNs, which had good tissue condition, no indentation, uniform thickness, and moderate length, were selected and boiled for 10 min. Then, the sample was cooled in cold water. After 5 min, the samples were wrapped in the fresh-keeping film for testing.

Before determination, the filter paper was used to absorb the water remaining on the noodles' surface. The noodle to be tested was placed on the stage of the material analyzer (Yingsheng Hengtai, China, TMS-Pro). The TPA texture characteristics assessed included hardness, elasticity, cohesiveness, and chewiness, as determined using a 50 mm flat probe. The speed before measurement, measurement speed, the initial force, the probe, and the compression rate were set as 30.0 mm/min, 30.0 mm/min, 0.1 N, 20.0 mm from the sample surface, and 60%, respectively.

### The microstructure of NASN

The microstructure of NASN was studied utilizing a scanning electron microscope (FEI, USA, NOVA 450) at 10 kV. We cut and fixed the short strip of NASN on the sample table with a diameter of 1.0 cm, sprayed gold coating, and then placed it under the electron microscope to observe and take photos at a magnification of 500 ×.

### Determination of the X-ray diffraction

X-ray diffractograms of dried NASNs were obtained with an X-ray diffractometer (Bruker, German, D8-Advance). The dried powder of NASN was tightly packed into the sample holder. Diffraction data were collected over an angular range from 4° to 70° (2θ) in a tube voltage of 40 kV, current of 30 mA, and a scanning rate of 4°/min.

### The stability and activity of NAs

The effects of the SN on the retention rate and the free radical scavenging rate of the NAs under the conditions of DRTS and WFS were studied using the FNs as a control. The changes in the retention rates of the NAs and the free radical scavenging rates of NASNs were observed both in DRTS condition with the water contents of two kinds of noodles of 13% and storage temperature of 20°C and in WFS condition with the water contents of 80% and storage temperature of – 18°C. The total storage time in the experiment was set at 2 months, and in the 1st month, the data collection was carried out every 5 days. In the 2nd month, data collection was done on the last day.

The dried SNs were crushed by a pulverizer and screened five times through 60 mesh to obtain powder samples. A solution sample of SN was prepared by adding 1.0 g of powder sample to 20.0 ml of 95% ethanol in a 50-ml beaker, shaking it until fully extracted, and filtering it for analysis. Then, 1.0 ml of the SN solution sample was transferred into a 10-ml volumetric flask, diluted with 95% ethanol to volume, and mixed. A 1-cm cuvette was used for TP analysis to analyze the absorbance value at 425 nm.

One gram of crushed sample was extracted by 10 ml of 60% ethanol using ultrasonic (Tianhua, China, KQC-2B) for 30 min. The extract was then centrifuged by a table freezing centrifuge (Jiawen, China, JW-2019HR) at 1,057 × *g* for 8 min. One milliliter of supernatant was aspirated, and 1 ml of Folin phenol reagent was put into a 10-ml test tube, followed by 2 ml of 12% Na_2_CO_3_ solution, which was then diluted to 10 ml with water and shaken well. The absorbance was measured at the wavelength of 765 nm for retention rate determination of curcumin after the reaction in the dark for 0.5 h at room temperature.

We accurately weighed 1.00 g of crushed sample, added about 2 ml of methanol, stirred the sample thoroughly, filtered the remaining residue, then added another 2 ml of methanol for cleaning. We then discarded the filtrate, extracted lycopene with a small amount of n-hexane, repeated the extraction step 2 or 3 times until the filtrate was colorless, transferred the filtrate into a 10-ml volumetric flask, shook it evenly with toluene to determine the lycopene extraction solution, and used a 1-cm cuvette to determine its absorbance value with toluene as blank under its maximum absorption wavelength of 485 nm.

### The determination of free radical scavenging rate

Preparation of sample solution: Dry/wet ground sample weighing 1.0/5.0 g (accurate to 0.001 g) was added to 10/20 ml of hot water (100°C), and kept at (100°C) for extraction for 45 min. The solution was filtered and the volume of the filtrate was fixed to 50 ml with deionized water.

### Detection process

About 0.5 ml of sample solution (0.0394 g DPPH dissolved in 500 ml absolute ethanol) was put into a test tube, and 0.5 ml of DPPH stock solution was then added and mixed well. The mixture of sample and DPPH was stranded for 30 min at room temperature for reaction, and then the absorbance was measured at 517 nm. The sample was replaced with 0.5 ml distilled water for the blank control group. The free radical scavenging rate was calculated according to formula 1.


(1) $$\begin{array}{l} \text{Free radical scavenging rate (\%)}\\ =\left[1-\frac{A_{1}-A_{2}}{A_{0}}\right]\times 100\% \end{array}$$

In the formula, the absorbance of the mixture of sample and DPPH, sample and ethanol and DPPH, and distilled water were stranded by letters A_1_, A_2_, and A_0_, respectively.

### Statistical analysis

All measurements were carried out in triplicate if not otherwise specified. Duncan's test was used to compare mean values at a significance level of *P* < 0.05 in the analysis of the TPA texture data using the SPSS 16.8 statistical analysis system (IBM Co. Ltd., USA). Microsoft Excel was used to summarize the experimental data, and origin 2019b software was used to draw figures.

## Results and discussion

### Effects of NAs addition amount on the quality of NASNs

**Figure 1:**
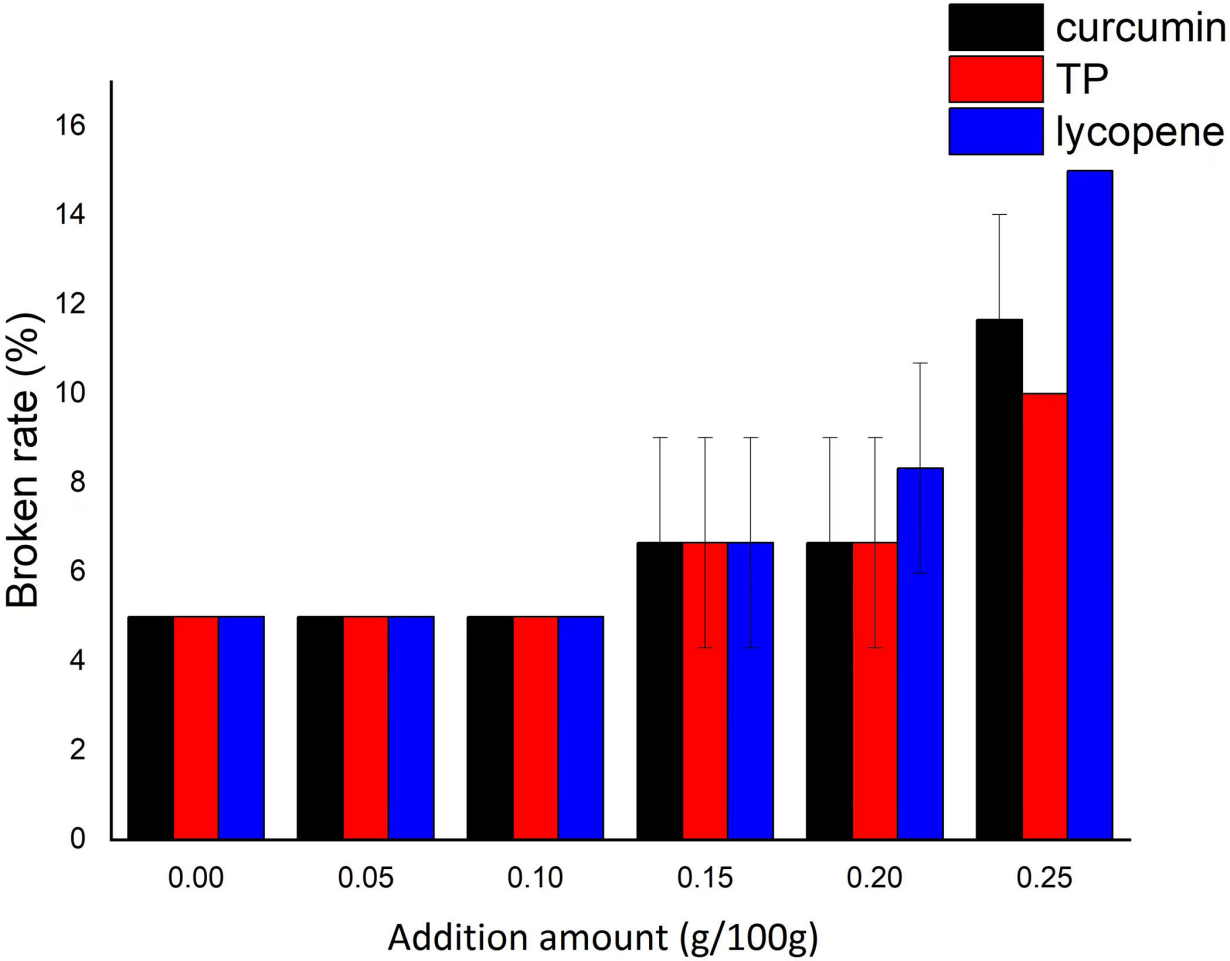
Effect of additional amount of NAs on the broken rate of NASNs.

The noodles quality of NASNs was evaluated using four indexes: broken rate, iodine blue value, sensory score, and TPA texture characteristics. The broken rate reflects the mechanical attributes of SN. The lower the broken strip rate, the greater the shear resistance and the better the boiling resistance (18). It can be found from Figure 1 that three kinds of NAs had a negligible effect on the broken rate of the NASN when the addition level of NAs was lower than 0.10/100 g. However, the broken rate showed an apparent upward trend when the addition level was increased from 0.15/100 to 0.25/100 g, indicating that the addition of NAs caused the internal polymerization force in SNs to weaken and had an adverse effect on the formation of the three-dimensional network structure of aging starch. Moreover, the broken rate caused by adding lycopene was significantly higher than that of the other two kinds of NASNs. However, the increase in the broken rate caused by adding TP was smaller than the other two, indicating that TP has the highest affinity with starch in the three NAs. Taking the broken rate of <10% as the acceptance standard (19), it can be concluded that the maximum amount of curcumin, TP, and lycopene in NASNs was 0.20/100, 0.25/100, and 0.20/100 g, respectively.

**Figure 2:**
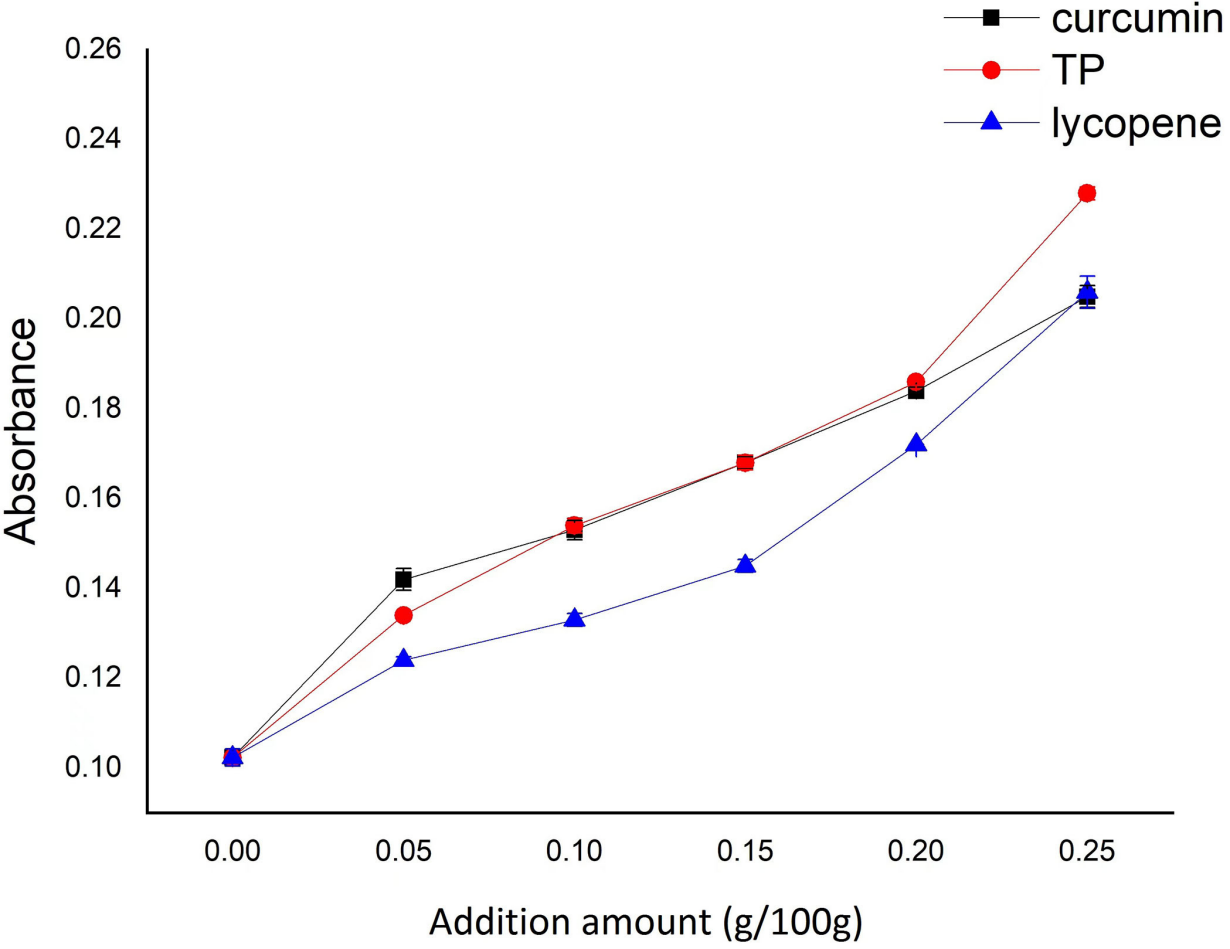
Effect of additional amount of NAs on iodine blue value of the soup of boiled NASNs.

The iodine blue value of the soup mainly indicates the extent of the soup paste rate. The higher the iodine blue value, the greater the soup paste rate (20). As demonstrated in Figure 2, the iodine blue value of the prepared NASNs increased with the addition of NAs. The most likely reason was that the addition of NAs interfered with the binding between starch and starch after the aging process and weakened the strength of the starch noodle, which is opposite to the structural changes caused by the addition of protein to starch noodle (21). The greater the amount of NAs, the higher the degree of interference. Moreover, due to the strong hydrophilicity of TP, it was easier to dissolve than the other two NAs when the noodles were heated and swollen in hot water. This trend will become more obvious with the increase of the additional amount, which demonstrated that the site exposure caused by the loss of more TP made the cross-linking between starch more vulnerable to the water, resulting in the fracture of the starch glycoside bond. The final performance was the increase of the iodine blue value of the soup.

**Figure 3:**
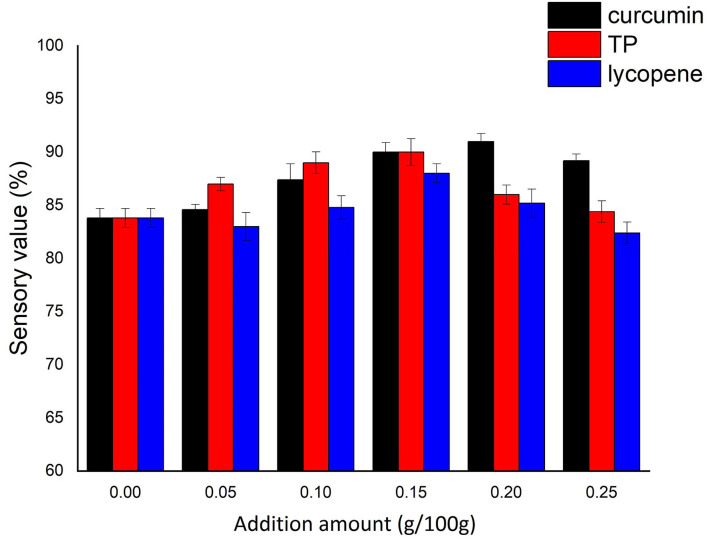
Effect of additional amount of NAs on the sensory value of NASNs.

The sensory scores of NASNs increased first and then decreased with the amount of NAs (Figure 3). These trends were consistent in the three kinds of NASNs. Due to the colors of the three NAs, the sensory scores were improved by the three NAs following low addition levels. However, when the addition amount is higher than 0.15/100 g for TP and lycopene or 0.20/100 g for curcumin, the sensory value of NASNs will show a downward trend. According to the experimental results, the optimal addition amounts of curcumin, TP, and lycopene in NASNs were 0.20/100, 0.15/100, and 0.15/100 g, respectively.

As seen in Table 1, adding three NAs can increase the hardness of NASNs. Compared with the other two NAs, lycopene had the highest increase in hardness, while TP had the lowest increase in hardness of NASNs. The chewiness of NASNs also showed a positive correlation with the amount of NAs, which was not associated with the kinds of NAs. Among the three NAs tested, the effect of TP on the chewiness of NASNs was significantly higher than that of the other two. However, the effects of adding curcumin and lycopene to FNs were quite different. Curcumin had little impact on the hardness and chewiness of FNs, while lycopene significantly improved their hardness and chewiness. The increase in the amount of NAs can also cause changes in the elasticity of the noodles. Additionally, the changes caused by various kinds of NAs were different. Adding curcumin and lycopene can reduce the elasticity of the SNs, which was similar to other research results in FN (22, 23). However, the changing trend of their elasticity obtained by adding TP was just the opposite, which should be related to the hydrophilicity of the NAs (23). Hydrophilic substances can combine more water in the colloid, increasing three-dimensional space's ability to resist deformation. Furthermore, Zhang et al. found through the tensile test that adding TP to starch-based food effectively improved its mechanical properties (24).

**Table 1:** Effect of addition amount of NAs on texture of NASNs.

**Addition amount (g/100 g)**	**Hardness (N)**			**Elasticity (mm)**			**Chewiness**		
	**Curcumin**	**TP**	**Lycopene**	**Curcumin**	**TP**	**Lycopene**	**Curcumin**	**TP**	**Lycopene**
0	33.7 ± 1.35^f^	33.7 ± 1.35^e^	33.7 ± 1.35^f^	1.20 ± 0.010^a^	1.20 ± 0.010^e^	1.20 ± 0.010^a^	25.6 ± 1.58^e^	25.6 ± 1.58^e^	25.6 ± 1.58^e^
0.05	36.4 ± 0.38^e^	37.4 ± 0.25^d^	39.6 ± 0.50^e^	1.19 ± 0.060^a^	1.23 ± 0.010^d^	1.11 ± 0.011^b^	27.0 ± 0.92^d^	29.7 ± 0.48^d^	27.8 ± 0.61^d^
0.10	37.5 ± 0.25^d^	38.4 ± 0.25^cd^	44.4 ± 0.10^d^	1.18 ± 0.150^ab^	1.27 ± 0.006^c^	1.09 ± 0.006^c^	28.3 ± 0.43^cd^	32.2 ± 1.52^c^	31.2 ± 0.39^c^
0.15	39.5 ± 0.30^c^	39.2 ± 0.10^bc^	46.3 ± 0.29^c^	1.17 ± 0.050^b^	1.30 ± 0.006^b^	1.08 ± 0.006^c^	29.5 ± 0.99^bc^	34.3 ± 1.09^b^	32.0 ± 0.55^bc^
0.20	42.0 ± 0.69^b^	39.7 ± 0.15^b^	51.1 ± 0.73^b^	1.14 ± 0.010^c^	1.33 ± 0.010^a^	1.06 ± 0.006^d^	30.8 ± 0.42^b^	35.3 ± 1.20^b^	34.4 ± 1.56^a^
0.25	47.8 ± 0.40^a^	41.9 ± 0.66^a^	54.3 ± 0.10^a^	1.11 ± 0.150^d^	1.33 ± 0.011^a^	1.00 ± 0.010^e^	33.8 ± 0.26^a^	38.9 ± 0.61^a^	33.3 ± 1.24^ab^

Note: The letters a–f indicate significant difference between the same column (*P* < 0.05), and the same letter represents no significant difference.

### The morphology of NASNs

**Figure 4:**
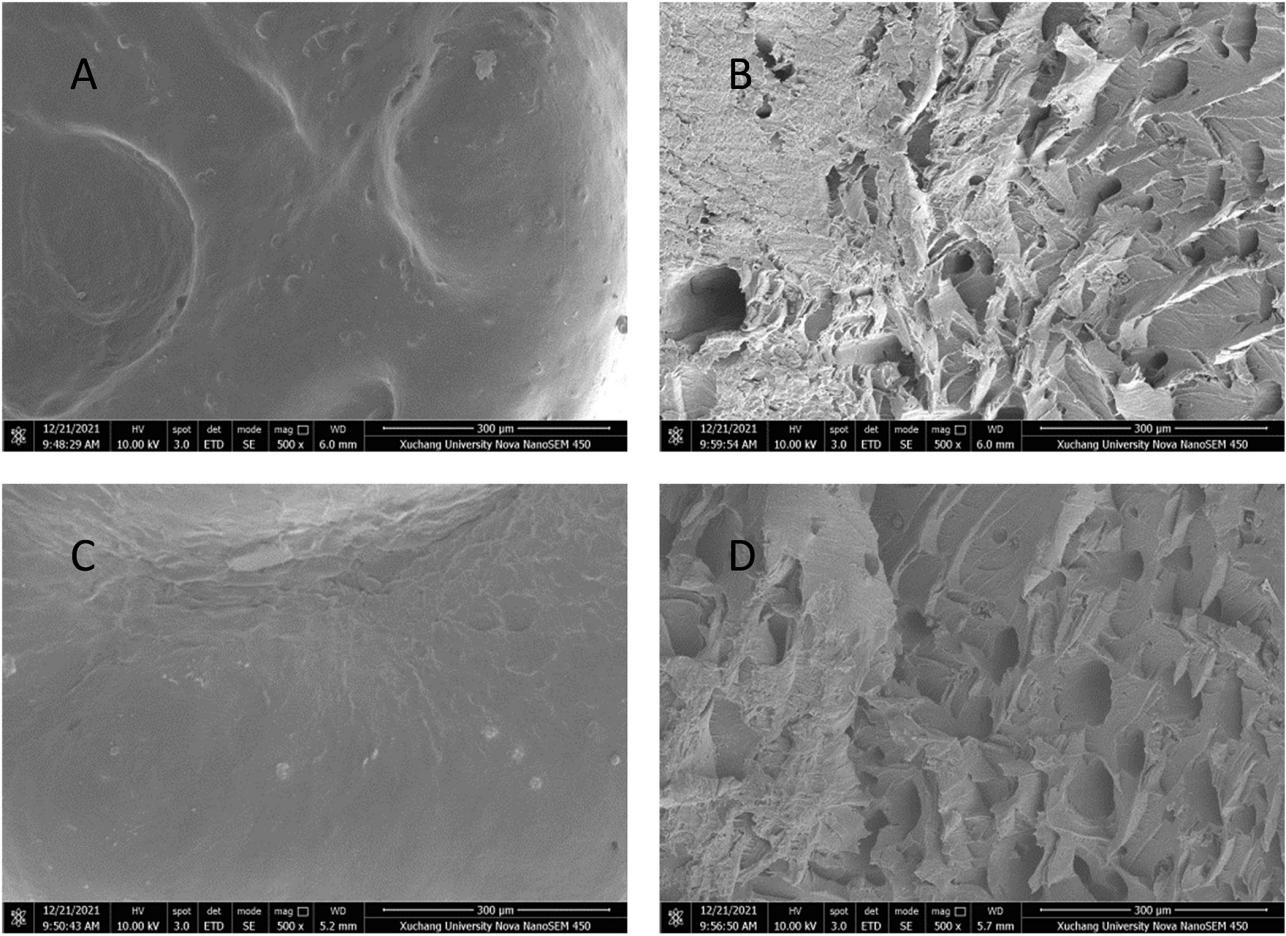
Micro-images of the cross-section of starch noodles containing no antioxidant **(A)**, 0.20/100 g of curcumin **(B)**, 0.15/100 g of TP **(C)**, and lycopene **(D)**, respectively.

The microstructures of the SNs prepared by adding different kinds of NAs are shown in Figure 4. The cross-section structure of the SN was compact and smooth and showed a continuous structure similar to a glassy state without any added NAs. Still, more holes in the cross-section appeared when adding NAs, indicating that the continuity of the starch gel was destroyed, and the small bubbles embedded in these holes were easy to spread and escape. Thus, the phenomenon of broken rates and cooking loss was more likely to occur in hot water cooking, which confirmed the results obtained from the analysis of the broken rates test and the iodine blue value test (25). The holes on the cross sections obtained by adding TP were smaller and more uniform than the other two NAs, indicating that water-soluble antioxidants were more likely to fuse with starch gel systems. Although there were some holes in the cross-section of the SNs added with curcumin and lycopene, their continuous network structures formed by starch gel still existed and maintained the integrity and basic mechanical properties of SNs to some extent. Specifically, with the addition of TP, the cross-sectional characteristic of SN was almost close to those of blank samples.

### X-ray characteristics

**Figure 5:**
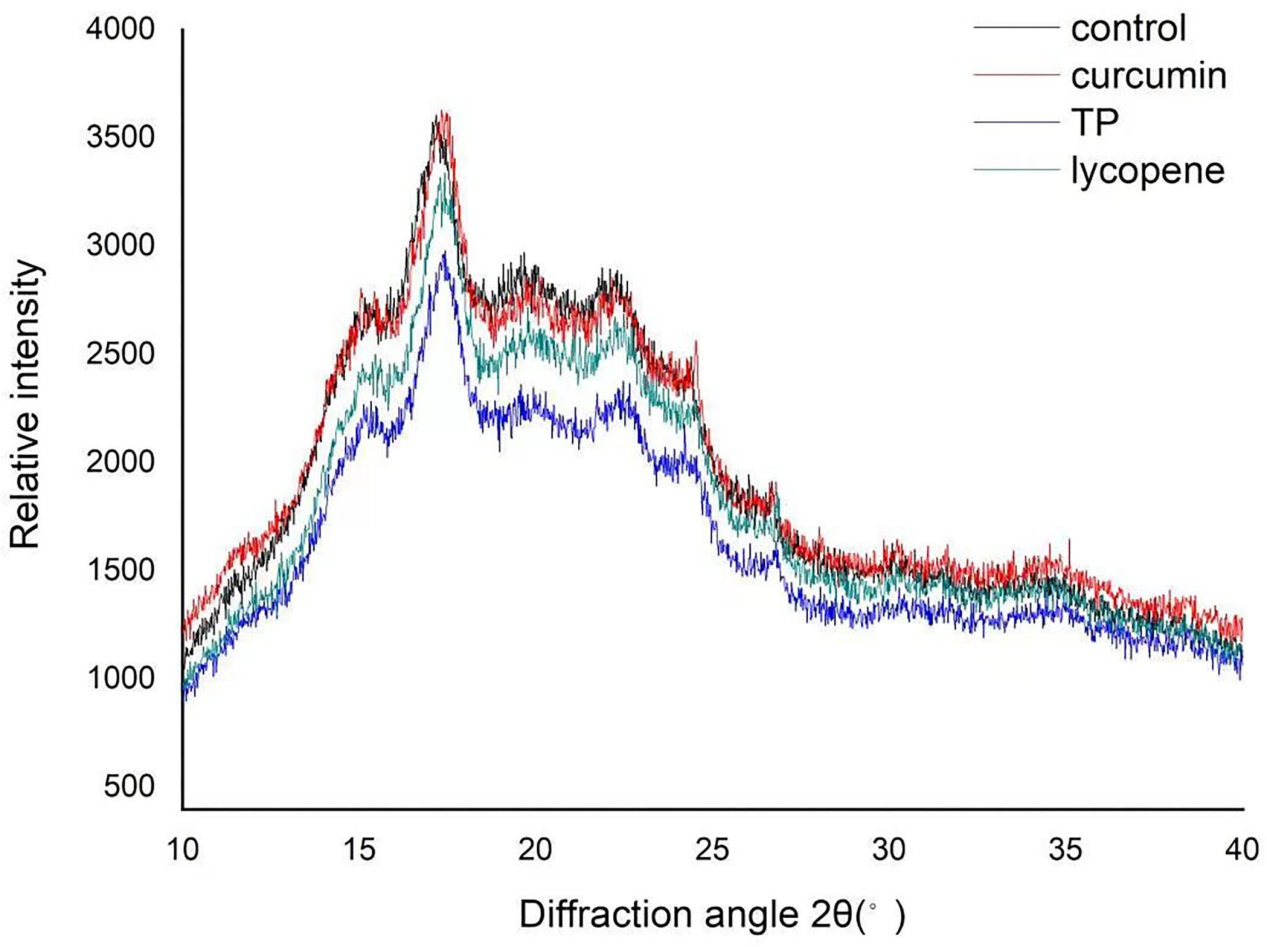
X-ray diffractogram of NASNs contained curcumin (red), TP (blue), lycopene (green), and SN without NAs (blank).

In the later stage of the production process of SNs, there was a cooling and aging process, which easily enabled the crystallization of starch, and the subsequent drying process would further increase its crystallinity (25). X-ray diffractograms of the SNs with the three kinds of NAs and control samples are presented in Figure 5. Three NASN samples showed peaks at the 2θ diffraction angles around 15, 17, 20, 23, and 26. The diffraction peaks at the 2θ diffraction angles around 15, 17, 20, and 23 are typical SPS crystals. The relative intensity of the diffraction peaks at 15 and 23 was significantly weakened. Still, peaks at 26 were not found in the SPS in previous studies, which showed that some kinds of crystal forms were extensively damaged, and some new forms occurred in the process (26). The most apparent diffraction peak at the 2θ diffraction angles was around 17, which indicated that a lower content of branched amylose and a higher content of amylopectin was contained in the four kinds of SNs (27, 28). Interestingly the samples added with curcumin showed pronounced diffraction peaks at the 2θ diffraction angles around 12, while the samples added with TP and lycopene showed prominent shoulder diffraction peaks. These diffraction peaks were not seen in the SNs without NAs, which demonstrated that the addition of these NAs affected the crystallization process of starch and produced a small proportion of new crystals in the NASNs.

### Retention rate changing of curcumin, TP, and lycopene

**Figure 6:**
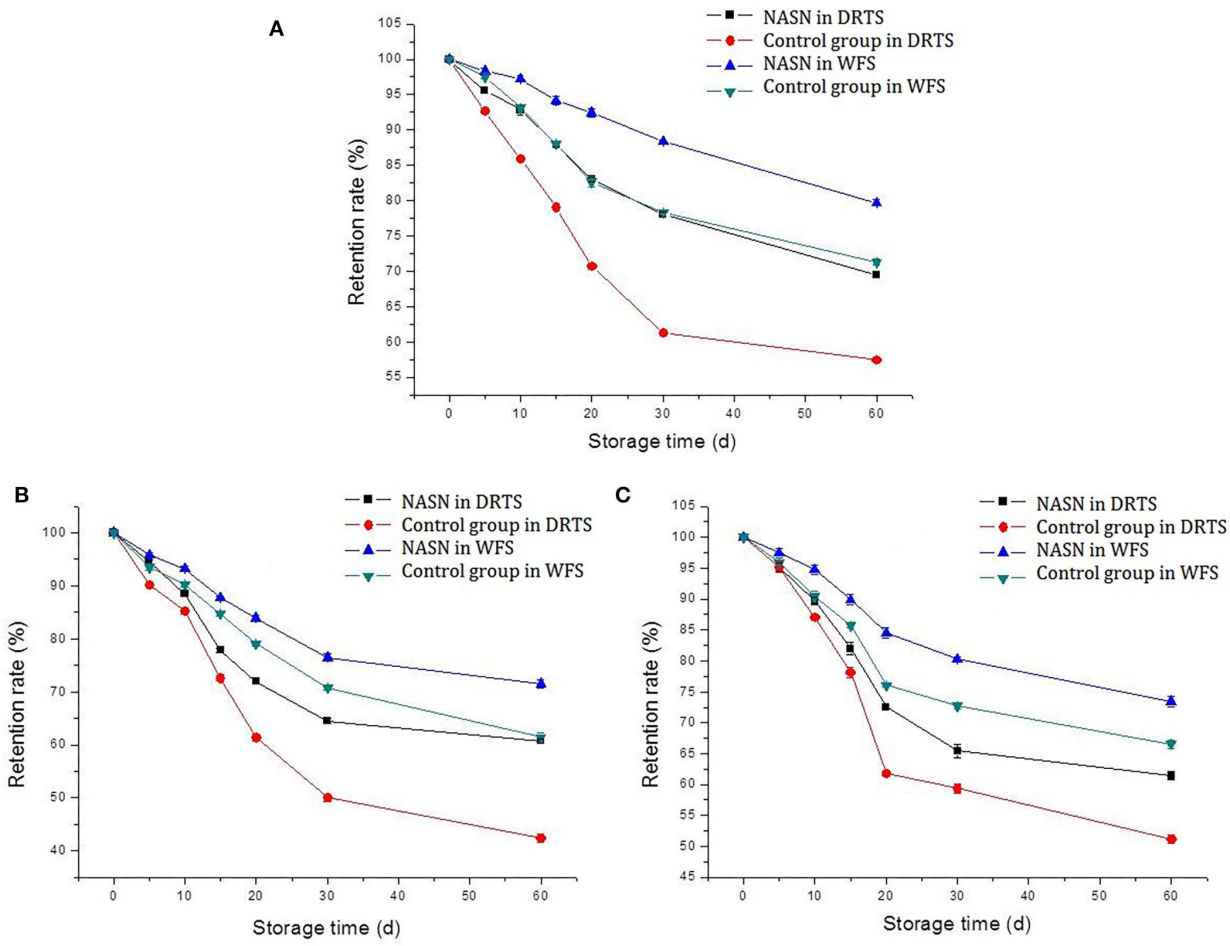
Retention rate changing of curcumin **(A)**, TP **(B)**, and lycopene **(C)** in NASNs and the control group in the condition of DRTS and WFS.

Three kinds of NASNs containing curcumin, TP, and lycopene were stored under both DRTS and WFS conditions for 60 days, and the natural antioxidant flour noodles (control group) were treated similarly. It was found that the retention rate trends of the three NAs in the NASNs and control group were similar, but the changing range differed (Figure 6). After 60 days of dry storage, the content retention rates of curcumin, TP, and lycopene were 69.54, 60.73, and 61.48%, respectively, which were significantly higher than those in the control group (57.45, 42.38, and 51.16%, respectively). Iqbal et al. (29) found that the retention rate of NAs, such as ascorbic acid, polyphenols, and carotenoids in dry, hot peppers, was between 77.4 and 87.3% after being packaged in natural jute or synthetic LDPE plastic bags and stored at ambient temperature for 5 months. Combined with the experimental results of this study, it showed that SNs could act as a protective package to protect NAs during storage to reduce the adverse effects of light or oxygen. The content retention rates of TP, curcumin, and lycopene in NASNs stored under WFS were 79.72, 71.54, and 73.43%, which were significantly higher than those in the control group (71.24, 61.40, and 66.57%, respectively). Addie A. Van der Sluis et al. (30) found that polyphenols and their antioxidant activity were relatively stable at low temperatures. The retention rates of the three studied NAs decreased rapidly when the storage time was <30 days, while the downward trends gradually flattened within 30–60 days. In contrast, the retention rates of the three NAs in the NASNs were significantly higher, and the decline rate was also slower than that in the control group both in the DRTS condition and WFS condition, indicating the protective ability of SN on NAs was stronger than that of FNs. The main reason was that the starch formed a concentrated elastic gel after high-temperature gelatinization and low-temperature aging (25). During drying, most of the moisture in the starch gel was removed, which promoted the retrogradation of starch and stabilized the product structure, thus forming a denser protective layer for NAs (31). But the starch content in flour was only about 60%, and there was no high-temperature gelatinization and low-temperature aging process of starch in the production process of FNs.

### The free radical scavenging rate

**Figure 7:**
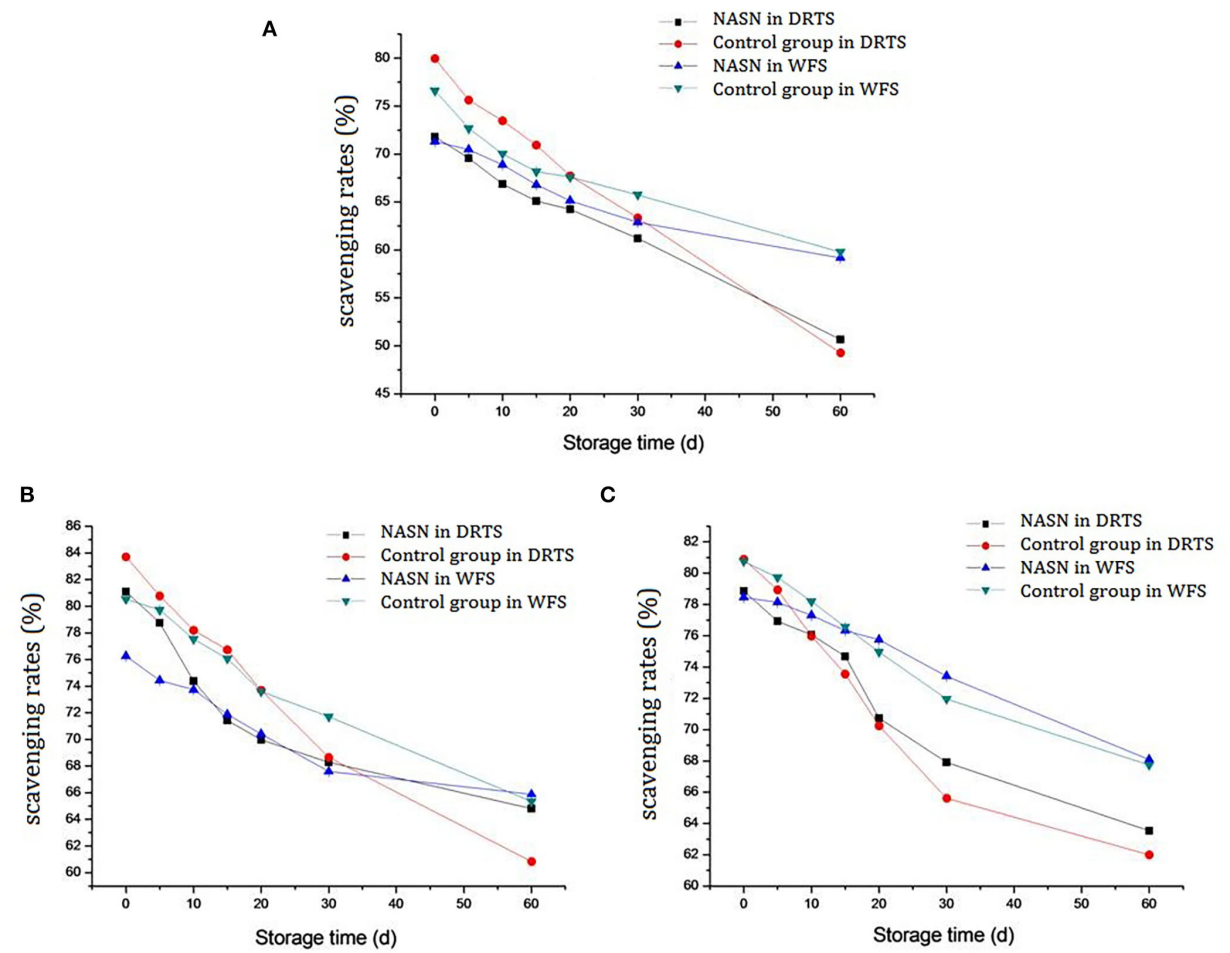
Free radical scavenging rate changing of curcumin **(A)**, TP **(B)**, and lycopene **(C)** in NASNs and the control group in the condition of DRTS and WFS.

After adding natural antioxidants to the noodles, the free radical scavenging rate by the DPPH method sharply increased (32). After 60 days of storage, the free radical scavenging rates of curcumin, TP, and lycopene in NASNs under DRTS conditions decreased by 21.12, 16.29, and 15.35%, respectively, which was significantly lower than that in the control group (30.24, 22.87, and 18.92%). Additionally, while under WFS conditions, they decreased by 12.14, 10.38, and 10.38%, respectively, which was also significantly lower than that of the control group (16.82, 15.19, and 13.02%) (Figure 7), indicating that the protective effect of SNs on the antioxidant activity of NAs was better than that of FNs. Although studies have shown that oil as a substrate can protect the activity of antioxidants, it is not enough to play this protective role because the oil content in FN was too low (<1%) (33). In addition, the heating procedure in the processing of NASNs was also conducive to maintaining the antioxidant function of the NAs during storage (34). Moreover, it could be seen intuitively from the figure that the free radical scavenging rates of NAs (red line with circle mark) decreased most significantly under DRTS conditions. In contrast, the downward trend of the same substances under WFT conditions (green line with triangle mark) was considerably alleviated, demonstrating that lower ambient temperatures positively affected the maintenance of antioxidant function.

## Conclusion

The effects of three kinds of NAs such as curcumin, TP, and lycopene, on sweet potato starch's structure and physicochemical properties of noodles were studied. Our results demonstrated that adding NAs changed the broken rate, iodine blue value, and elasticity. However, the sensory quality, nutrition, and antioxidant function of NASN were improved if the curcumin, TP, and lycopene were 0.20/100, 0.15/100 g, and 0.15/100 g, respectively. Moreover, SN was more conducive to maintaining NAs and their functional activities. Therefore, these findings indicate that NAs have good application values in SNs.

## Data availability statement

The original contributions presented in the study are included in the article/supplementary material, further inquiries can be directed to the corresponding author/s.

## Author contributions

The planning of the research was done by WG, XG, and JH. The experiments were performed by LF, YW, GL, and ZC. All the writers have contributed in the research work and the write-up of this article. All authors contributed to the article and approved the submitted version.

## Funding

We acknowledge the funding support from the Major Science and Technology Projects in Henan Province, China (ID No. 201300110300), Key Scientific Research Projects of Universities in Henan Province (ID No. 22B550017), and the Training Program for Young Backbone Teachers in Colleges and Universities in Henan Province, China (ID No. 2019GGJS216).

## Conflict of interest

The authors declare that the research was conducted in the absence of any commercial or financial relationships that could be construed as a potential conflict of interest.

## Publisher's note

All claims expressed in this article are solely those of the authors and do not necessarily represent those of their affiliated organizations, or those of the publisher, the editors and the reviewers. Any product that may be evaluated in this article, or claim that may be made by its manufacturer, is not guaranteed or endorsed by the publisher.

## Abbreviations

NASN: natural antioxidant starch noodle
NA: natural antioxidant
SPSN: sweet potato starch noodle
TP: tea polyphenols
SN: starch noodle
SPS: sweet potato starch
FN: flour noodle
WFS: wet and freezing storage
DRTS: dry and room temperature storage
GI: glycemic index
SEM: scanning electron microscope.
